# A four-part guide to lung immunology: Invasion, inflammation, immunity, and intervention

**DOI:** 10.3389/fimmu.2023.1119564

**Published:** 2023-03-31

**Authors:** Indiwari Gopallawa, Ruchika Dehinwal, Vaibhav Bhatia, Vikramsingh Gujar, Narendra Chirmule

**Affiliations:** ^1^ Clinical Pharmacology & Safety Sciences, Biopharmaceuticals R&D, AstraZeneca, Gaithersburg, MD, United States; ^2^ Department of Microbiology, Division of Infectious Disease, Brigham Women’s Hospital, Harvard Medical School, Howard Hughes Medical Institute, Boston, MA, United States; ^3^ R&D Department, Lamark Biotech, Vellore, India; ^4^ Department of Anatomy and Cell Biology, Oklahoma State University Center for Health Sciences, Tulsa, OK, United States; ^5^ R&D Department, SymphonyTech Biologics, Philadelphia, PA, United States

**Keywords:** lung, immunity, inflammation, invasion, intervention

## Abstract

Lungs are important respiratory organs primarily involved in gas exchange. Lungs interact directly with the environment and their primary function is affected by several inflammatory responses caused by allergens, inflammatory mediators, and pathogens, eventually leading to disease. The immune architecture of the lung consists of an extensive network of innate immune cells, which induce adaptive immune responses based on the nature of the pathogen(s). The balance of immune responses is critical for maintaining immune homeostasis in the lung. Infection by pathogens and physical or genetic dysregulation of immune homeostasis result in inflammatory diseases. These responses culminate in the production of a plethora of cytokines such as TSLP, IL-9, IL-25, and IL-33, which have been implicated in the pathogenesis of several inflammatory and autoimmune diseases. Shifting the balance of Th1, Th2, Th9, and Th17 responses have been the targets of therapeutic interventions in the treatment of these diseases. Here, we have briefly reviewed the innate and adaptive i3mmune responses in the lung. Genetic and environmental factors, and infection are the major causes of dysregulation of various functions of the lung. We have elaborated on the impact of inflammatory and infectious diseases, advances in therapies, and drug delivery devices on this critical organ. Finally, we have provided a comprehensive compilation of different inflammatory and infectious diseases of the lungs and commented on the pros and cons of different inhalation devices for the management of lung diseases. The review is intended to provide a summary of the immunology of the lung, with an emphasis on drug and device development.

## Introduction

Lungs are important respiratory organs involved in providing oxygen to the body. Every day, the lungs interact directly with a myriad of different-sized particles from the environment. Most of these particles are filtered through the mucosal barriers present in the upper respiratory tract. These barriers thus efficiently protect the body from pathogens, allergens, and various foreign bodies. Large particles are removed by physical barriers and forces such as coughing and sneezing, triggered by the muscular-nervous system. Smaller particles are entrapped, degraded, and processed by epithelial and a battery of cells in the mucosal lining of the trachea. Interactions of these external substances with specific Toll-like receptors present on the inner epithelium of the respiratory tract and phagocytosis of these substances activate the innate immune system (1). This system includes the induction of anti-microbial peptides, chemokines, and cytokines. These mediators of the innate immune system subsequently recruit and activate a complex array of adaptive immune responses, which have been classified based on the kind of cytokines into Th1, Th2, Th9, and Th17 responses. The precise regulation, i.e., activation, persistence, and termination of these responses involve activation of transcription factors through a variety of signal transduction pathways.

In this review, we have summarized various aspects of pulmonary immune homeostasis mediated through regulated activation of innate and adaptive immune responses. We have elaborated on the pathogenesis of inflammatory autoimmune diseases and infectious diseases. Further, we have highlighted the advances in novel therapies and drug delivery devices that can help in developing drugs to treat lung diseases.

## Lung homeostasis

**Figure 1:**
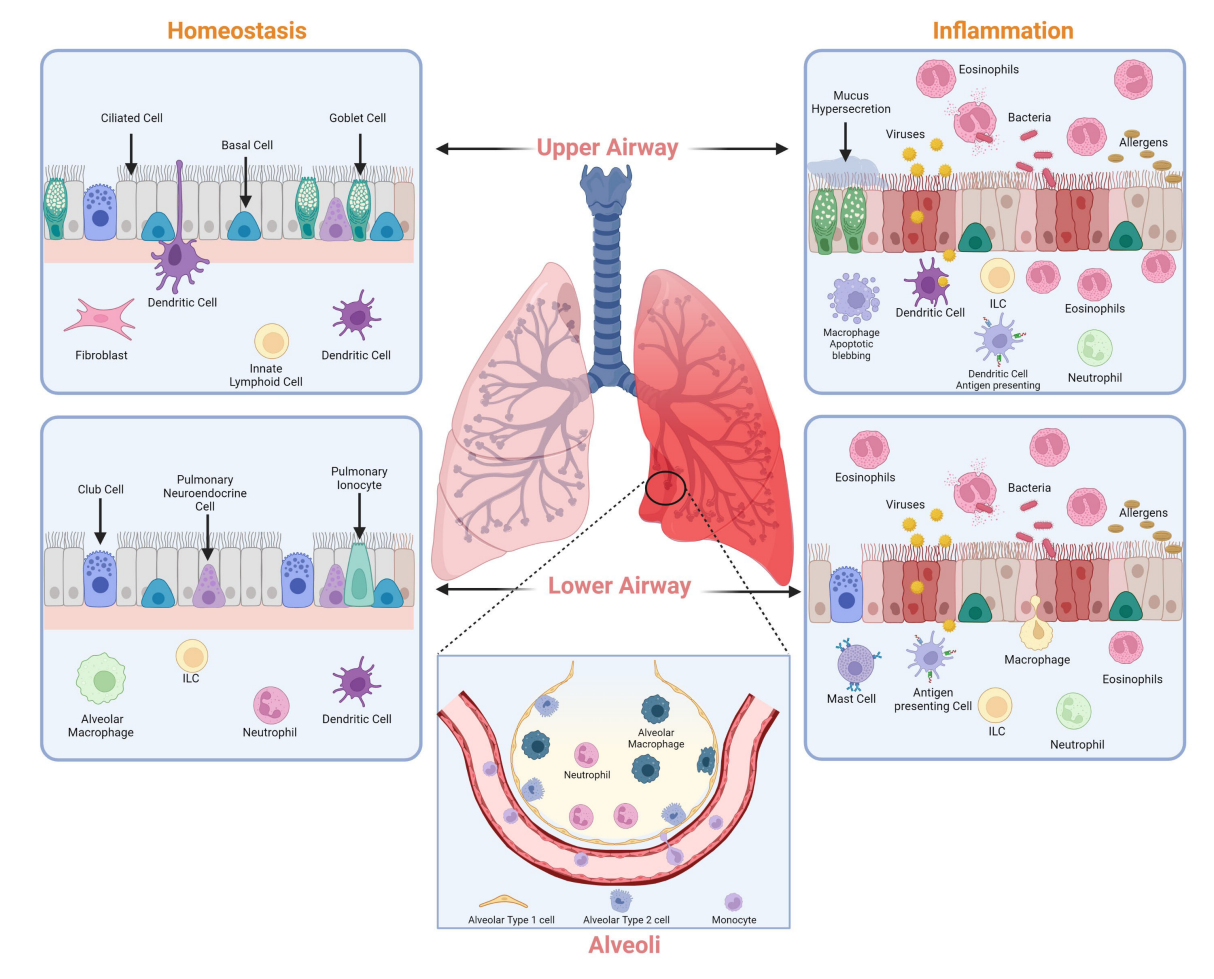
Major cell types of the lung. Cell types that contribute to the maintenance of lung homeostasis (left) and lung inflammation (right) in the upper and lower airways. Image created with Biorender.com.

Homeostasis is a state of balance. Homeostasis within the lung is maintained by harmonious coordination of different immune pathways, which includes the active participation of alveolar epithelial cells (AECs) and macrophages (**Figure 1**). AEC I and AEC II make up most of the mature alveolar epithelium and AEC I covers almost 99% of the lung surface area which is crucial for gas exchange and protection against a myriad of inhaled external particles. Alveolar epithelium is developed by organized processes that begin in the ventral foregut endoderm during development. Interactions between the foregut epithelium and its associated mesenchyme promote the formation of a functioning respiratory system and its compartmentalization along the proximal-distal axis (2, 3). The lung epithelium is a physical barrier between the lumen and underlying submucosa, protecting the lung tissue from bacteria, viruses, allergies, and other harmful substances. Besides acting as a physical barrier, AECs are crucial in the lung’s immune response. Although both AEC I and AEC II are essential in airway defense mechanisms, AEC II is more immunologically active (4). AECs produce several immune factors such as cytokines and chemokines that are responsible for immune cell activation and differentiation and act as antigen-presenting cells for specific T cells (5–7). Other cells, such as macrophages, also participate in the normal physiological functions of the lung and are involved in some acute and chronic diseases (8, 9). There are two types of lower respiratory tract macrophages: alveolar macrophages (AMs) in the alveoli and interstitial macrophages (IMs) in the interstitium. Both kinds of macrophages produce robust responses to a wide range of stimuli. AMs are innately suppressive and play an essential role in maintaining immunological homeostasis and host defense.

In contrast to AMs, IMs have a regulatory function within the lung tissue (10). While AMs are regulated through non-specific defenses such as phagocytosis, tumor necrosis factor-alpha (TNF-α), and interferon gamma (IFN- γ), IMs have a higher proclivity for releasing cytokines related to the adaptive immune response (11). Overall, both AECs and macrophages work together to maintain the decorum in the lung under normal conditions and contribute to regulating the immune system.

A detailed understanding of the pathogenesis of inflammatory diseases and lung infections has given insights into the multifactorial components of the immune system in regulating lung functions. We have reviewed these two conditions and listed the immunological pathways that contribute to the immunopathogenesis of various inflammatory diseases and infections. There are several learnings of immunological pathways from these diverse pathologies that have yielded novel therapies. We have outlined the advances in delivery devices for therapeutic interventions that have been developed to treat lung diseases.

## Immune responses in the lung

Every day, the respiratory system, processes 10,000–12,000 L of air. Due to this, the lung epithelium is exposed to several particulates mixed with a complex mixture of gases (12). While most of the air includes safe environmental components, sometimes, the inhaled air contains harmful microbes and allergens. The conducting airways and gas exchange surfaces must deal with such hazardous substances and quickly react to protect the host. To achieve protection, the respiratory system must coordinate with the pulmonary immune system to protect the host from a plethora of threats. Less virulent microbes are generally obliterated by primary defenses like mucociliary clearance and alveolar macrophages in the respiratory tract. On the contrary, more virulent microbes and a large number of microbes need to be eliminated by inflammatory processes initiated by the innate immune system (13). The pulmonary immune system is composed of innate and adaptive immune systems. The innate immune system is the first line of defense against pathogens and sterile threats. Innate protection is primarily provided at the cellular level by coordinated actions of the airway and alveolar epithelial cells, resident macrophages, recruited neutrophils, and monocytes, which respond rapidly to inhaled materials (**Table 1**). The initial checkpoint is provided by epithelial cells, dendritic cells, and alveolar macrophages during antigen infiltration, which induces a proinflammatory downstream immune response. The innate defense in the respiratory tract is coordinated by several components such as lysozymes, lactoferrin, secretory leukoprotease inhibitor, proinflammatory cytokines, chemokines along with antimicrobial secretory IgAs (sIgA), defensins and cathelicidins (29–31) The intercellular communication between the airway and the immune cells is facilitated by lipid mediators, complement factors, chemo-attractants, and chemokines. The biological function of chemokines is relayed by different cytokines, including several interleukins and thymic stromal lymphopoietin (TSLP) (32, 33). These substances, secreted by local airway cells, generate customized immunological responses.

**Table 1:** Cells involved in primary Immune response of the lung.

Cells	Function	Receptors	References
**Airway Epithelial cells**	- Barrier protection - Maintain fluid balance - Regulate mucus and surfactant production - Initiation of immune response *via* cytokine, chemokine, and growth factor production	- Toll-like receptors - RIG-I-like receptors - Nod-like receptors (NLR) - C-type lectin receptors - Bitter and sweet-taste receptors	(13–16)
**Neutrophils**	- Link between Innate and adaptive immunity - Clearance of exogeneous pathogens - Clearance of endogenous cell debris - Involved in pathogenesis of respiratory diseases	- G-protein-coupled receptors - Fc-receptors - Adhesion receptors - Cytokine receptors - Innate immune receptors	(13, 17–19)
**Alveolar Macrophages**	- Phagocytic and antigen presenting cell - First line of defense against pollutants and pathogenic microbes - Specialized in detecting environmental signals - Communicate with other cells to regulate lung homeostasis	- Scavenger receptors - Fc receptors - G-protein-coupled receptors - Integrins - CD-14 - Toll-like receptors	(13, 20, 21)
**Innate Lymphoid cells** **(ILCs)**	- Lack antigen-specific receptors - Protect lung by producing IFN- γ, IL-5, IL-13, IL-17 and IL-22 - Induce airway hyperinflammation - Promote allergic inflammation - Blocks extracellular bacterial infections	- IL-2Rα (CD25), - IL-7Rα (CD127), - IL7Rγ (CD132)	(13, 22–24)
**Mucosal-Associated Invariant T Cells** **(MAIT)**	- Play role in pulmonary homeostasis and innate immunity to pathogens - Recognize microbial metabolites *via* MHC-I molecule - Directly associated with the pathogenesis of lung diseases - Involved in immune response against SARS-CoV-2	- C-type lectin receptors (CD161) - Chemokine receptors (CCR9, CXCR6) - Cytokine receptors (IL-12R, IL-18Rα, IL-17Rα)	(13, 25–27)
**Dendritic Cells**	- Initiation of adaptive immune response and inflammatory process	- TLR-7 and 9 - Fcγ receptors (FcγR) II - Blood dendritic cell antigen-2 (BDCA-2) - Dendritic cell immunoreceptor (DCIR)	(28)

The respiratory adaptive immune system consists of cellular components like T lymphocytes, B lymphocytes, and antigen-presenting cells, along with soluble mediators like cytokines, chemokines, lactoferrin, defensins and sIgAs (34). Following the mechanisms of the innate immune system, the lungs are further protected by the mechanisms of the adaptive immune system against various external and internal threats such as pathogens entering alveoli and microorganisms inside lung cells. The most common immune cell types in the lungs are macrophages and dendritic cells, which are innate cells, and CD4+ and CD8+T lymphocytes, constitute adaptive immune cells (14). Most lung macrophages are AMs and are essential for infection resistance. However, they have been linked to dysregulations in lung inflammation and fibrosis (8). T cells are primed and activated in lymphoid organs before migrating to the lung to respond to respiratory infections. They survive as memory T cells following pathogen clearance. The lung was previously found to have prominent memory T cell populations, which were first assumed to constitute a migratory surveillance population. However, a bulk of lung T cells are now known to survive as noncirculating tissue-resident memory T cells (TRMs). Pools of memory T cells are created throughout a respiratory viral infection and remain for the span of the animal’s life. These cells feature different cytokine production profiles, minimal need for co-stimulation, reduced sensitivity to apoptosis, and persist at high frequency compared to their naïve progenitors. The spleen and draining lymph nodes are secondary lymphoid organs with a significant number of memory cells (15). The functions of T cells especially CD4+ T lymphocytes and how cytokines produced by these T cells play a role in lung diseases such as IPF, COPD, and asthma are described in the section below.

## Inflammation

Inflammation is defined as the response of the immune system to injury (35). Inflammation is favorable to the host under normal circumstances. However, an abnormal immune response(s) in the airway can lead to chronic inflammatory responses, contributing to a variety of lung diseases. In this review, we have summarized the role of CD4^+^ T cells and AEC apoptosis which is a prominent observation in lung biopsies of patients with idiopathic pulmonary fibrosis (IPF). Further, we discuss the role of inflammation in chronic obstructive pulmonary disease (COPD) and asthma. The role of inflammation in IPF is considered somewhat controversial and reviewed extensively elsewhere (36). Additionally, we summarize currently available FDA-approved drugs for IPF, asthma, and COPD (**Table 2**).

**Table 2:** Summarizes FDA approved drugs to treat fibrosis, Asthma, and COPD.

Disease	Drug	Mechanism of Action	FDA approval	Reference
**Fibrosis**	Pirfenidone	Small molecule inhibitor that inhibits both the production and activity of TGF-β. Decreases collagen synthesis.	2014	(37)
	Nintedanib	Small molecule inhibitor of the receptor tyrosine kinases such as PDGF receptor, FGF receptor and vascular endothelial growth factor receptor.	2014	(38, 39)
**Asthma**	Omalizumab	Monoclonal antibody that binds to free circulating IgE and inhibits receptor interaction.	2003	(40)
	Mepolizumab	Humanized, IL-5 antagonist that reduces IL-5 signaling and activation of eosinophils.	2015	(41)
	Reslizumab	Monoclonal antibody that targets IL-5.	2016	(42)
	Benralizumab	Monoclonal antibody that binds to IL-5R hindering the binding of IL-5 to its receptor and thereby inhibiting eosinophil differentiation.	2017	(43)
	Tezepelumab	Monoclonal antibody that binds to TSLP and block the upregulation of Th-2 cytokines.	2021	(44)
**COPD**	Budesonide and formoterol fumarate dihydrate	Reduces inflammation and leads to airway dilation.	2009	(45)
	Roflumilast	A selective phosphodiesterase 4 (PDE4) inhibitor to reduce airway inflammation.	2011	(46)
	Umeclidinium/vilanterol	A muscarinic antagonist and beta2 agonist in combination that leads to airway dilation.	2013	(47)
	Olodaterol/tiotropium	Olodaterol is an anticholinergic agent that prevents bronchoconstriction and tiotropium leads to bronchodilation.	2015	(48)
	Glycopyrrolate, and formoterol fumarate	A muscarinic antagonist and β_2-_agonist that causes bronchodilation.	2016	(49)
	Aclidinium bromide and formoterol fumarate	Muscarinic antagonist and a β_2_-agonist that leads to bronchodilation.	2018	(50)
	Yupelri (revefenacin)	A muscarinic antagonist that causes bronchodilation and improve lung function.	2018	(51)
	Budesonide, *glycopyrronium, formoterol* fumarate	A triple therapy that consists of an inhaled corticosteroid, anticholinergic, and β_2_-agonist that improves lung function.	2020	(52)

### Idiopathic pulmonary fibrosis

Fibrosis is a highly dynamic process, and it can affect any organ including the skin, lungs, liver, and kidney. IPF is defined as an excessive deposition of extracellular matrix (ECM) proteins in the lung tissue, which makes the lungs unable to transport oxygen into the bloodstream efficiently and often leads to lung failure. Although the etiology of IPF is unknown, several risk factors such as cigarette smoking, occupational factors, genetic polymorphisms, and environmental exposure can contribute to the development of IPF. IPF is a clinically heterogeneous disease with an overall prognosis being very poor; typically, it has a median survival of 3–4 years (35, 53). Repetitive injury to the alveolar epithelium, failure to reepithelization, increased AEC apoptosis, and induction of growth factors and profibrotic cytokines such as transforming growth factor-β1 (TGF-β1), TNF-α, platelet-derived growth factor, and connective tissue growth factor (CTGF) are implicated in the progression of IPF (54). In addition to AEC apoptosis, fibroblast differentiation, accumulation of myofibroblasts, and excessive collagen deposition in the alveolar space promote the progression of fibrosis (55, 56).

**CD4+ T cells in IPF**: One of the main challenges in lung fibrosis is that the exact molecular and cellular mechanisms underlying the disease remain largely unknown. However, damage to the airway epithelium (apoptosis) is considered as an early pathogenic event, with an irregular repair process by epithelial-mesenchymal interactions, rather than an inflammatory response (57). Although over the several decades the role of inflammation has been questioned in fibrosis (58), data from IPF patients and experimental animal models have shown a prominent role of T helper cell type-2 (Th2) cells in IPF (59). It has been shown that each of the major Th2 cytokines such as IL-4, IL-5, and IL-13 contribute to the regulation of tissue remodeling and subsequent fibrosis. In-vitro studies have shown that stimulation with IL-4 increased collagen production in murine fibroblasts (60). Moreover, IL-4 concentrations were increased in the bronchoalveolar lavage fluids from patients with idiopathic pulmonary fibrosis (61). Along similar lines, IL-4 has been shown to induce periostin which may play a role in fibrosis (62).

IL-13 is another Th-2 cytokine that has been shown to play a key role in lung fibrosis and increased expression of IL-13 was repeatedly reported in IPF. Overexpression of IL-13 augmented subepithelial airway fibrosis in the mouse lung (63). Studies done in animal models have demonstrated that treating with anti-IL-13 antibodies significantly decreased aspergillus fumigatus-induced collagen levels in the lung (64). In a different study, it was demonstrated that IL-13 deficient mice were unaffected by radiation-induced fibrosis compared to wild-type mice (65).

In addition to IL-4 and IL-13, it has been observed that IL-5 can also play a role in tissue remodeling in several diseases including pulmonary fibrosis (66). It was demonstrated that IL-5 levels were increased in the lungs of bleomycin-treated mice (67). Further, it was shown that anti-IL-5 antibody caused a significant reduction in lung eosinophils and fibrosis in a bleomycin-induced pulmonary fibrosis mouse model (68). Given the role of IL-4, IL-5, and IL-13, it is crucial to identify other cytokines and receptors that may contribute to the aberrant cellular mechanisms in the IPF lung.

**AEC apoptosis in IPF**: The respiratory epithelium is a physical and functional barrier, shielding the underlying lung microenvironment. It plays a crucial role in the clearance of pathogens and harmful bacteria (69). Apoptosis, or programmed cell death, is a vital biological process in the maintenance of tissue homeostasis (70). However, in recent years, seminal works have supported the notion that excessive apoptosis is linked to diseases such as IPF, emphysema, and acute lung injury (71). It has been shown in a variety of animal models that blockade of AEC apoptosis reduces subsequent fibrosis (72). A broad-spectrum caspase inhibitor has been shown to decrease caspase-1 and caspase-3 activity, reducing the number of apoptotic cells. This led to a reduction in lung collagen in a bleomycin-induced mouse model (73). At a cellular and molecular level, there are many signaling pathways and factors that contribute to AEC apoptosis, such as the angiotensin (ANG) system. Several studies have demonstrated that manipulation of the ANG system can control apoptosis in the alveolar epithelium (74). Therefore, protecting AECs from apoptosis and sustaining their function might be an effective therapeutic strategy to decrease lung injury.

**Current treatment strategies for IPF**: Recent research contains a wealth of information on cellular and molecular signaling mechanisms in IPF, and we now have a better understanding of its pathophysiology. Specifically, IPF is no longer considered a pro-inflammatory disorder, but rather it is the result of a fibroproliferative environment and aberrant wound healing mechanisms (75) Currently, two antifibrotic therapies are available for IPF patients: pirfenidone and nintedanib. Pirfenidone has been recognized for its antioxidant and antifibrotic effects (76) is a tyrosine kinase inhibitor that inhibits fibroblast proliferation and differentiation and improves lung function (77). Given the heterogeneity of IPF, targeting multiple signaling pathways and multiple cell types may offer novel therapeutics to patients.

### Chronic obstructive pulmonary disease

COPD is a disease with high morbidity and mortality rates. It is characterized by chronic inflammation that leads to poorly reversible airway obstruction (78). The most common risk factor for the development of COPD is cigarette smoke. However, other harmful environmental factors such as exposure to air pollutants might contribute to COPD. Patients with COPD have significant levels of circulating inflammatory markers, demonstrating the presence of systemic inflammation (79). Persistent airway inflammation in COPD contributes to airway remodeling, loss of small airways, and emphysema (80). Emphysema is a major pathological change of COPD, which is characterized by the enlargement of alveolar airspaces due to the destruction of the alveolar epithelium (81).

**Figure 2:**
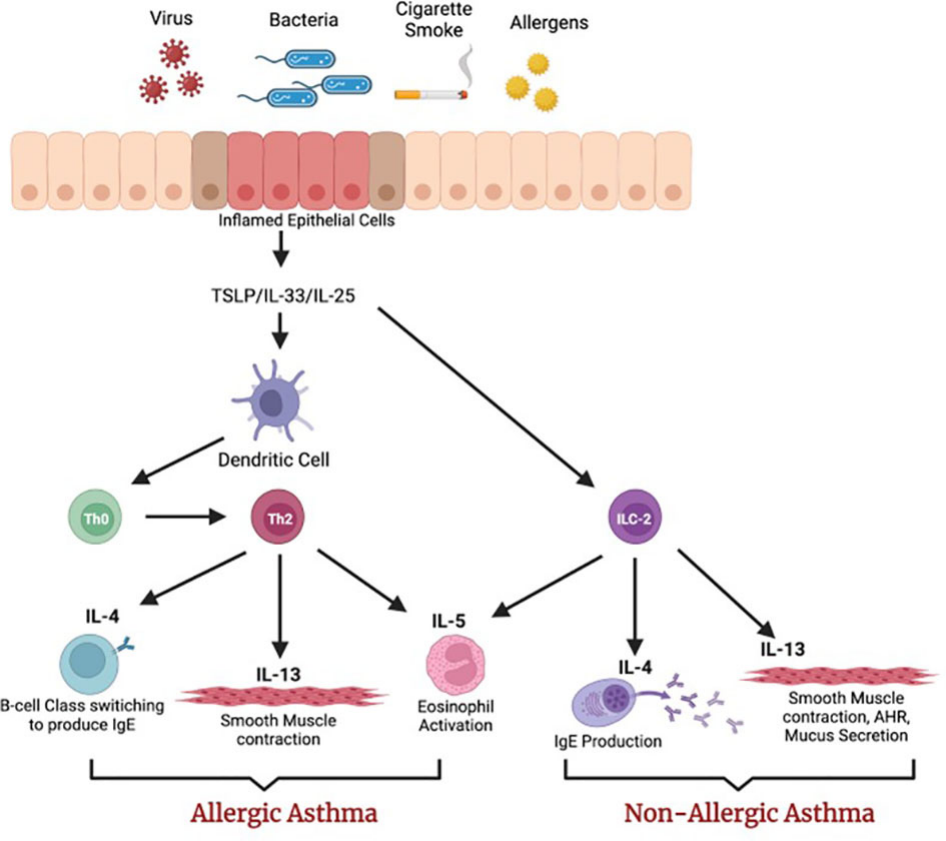
The role of the alarmins in the lung epithelium. Exposure to allergens, viruses, and bacteria releases cytokines from the lung epithelium that can activate dendritic cells (DC) and innate lymphoid cells (ILC-2) that belong to the innate immune system. DCs differentiate naïve T cells into Th2 cells that can secrete IL-4, IL-13, and IL-5. These cytokines play a role in airway remodeling. Similarly, ILC-2s secrete Th2 cytokines. Image created with BioRender.com.

**Inflammation in COPD:** The pathogenesis of COPD is multifaceted and the underlying cellular and molecular mechanisms remain poorly understood. In COPD, the inflammatory response involves both innate and adaptive immunity. The predominant form of COPD is neutrophilic inflammation, which correlates with the rate of decline in lung function (82, 83). In addition to the well-known augmented influx of neutrophils in COPD, aberrant clearance of neutrophils possibly contributes to the dysregulated inflammation in COPD (84). Aberrant clearance of neutrophils might be a result of defective phagocytic responses in alveolar macrophages. It was demonstrated that cigarette smoke could hinder the clearance of neutrophils, inducing pro-inflammatory cytokines such as TNF-α (85). According to a recent study by Borges et al., the administration of TNF-α weakened the removal of apoptotic cells from the lungs in a mouse model of LPS-induced neutrophilic inflammation (86). Even though neutrophil-associated inflammation is the most common inflammatory phenotype in COPD, 10%–40% of COPD patients demonstrate increased eosinophilic inflammation (87). Additionally, it was shown that airway *β*-defensin-1 levels were significantly elevated in the sputum of COPD patients (88) whereas secretory IgA (sIgA) levels were markedly reduced due to altered epithelial cell differentiation in the airways of COPD patients (89) Following exposure to cigarette smoke, pollutants, and microbes, alarmins such as TSLP, IL-33, and IL-25 are released from the epithelium. Following T-cell differentiation, Th-2 cells produce IL-4, IL-5, and IL-13, all of which contribute to an inflammatory environment (**Figure 2**) (90). Induction of IL-4 can lead to an increase in total IgE levels in eosinophilic COPD, which might contribute to a decline in lung function (91).

**Current treatment strategies for COPD:** Although current therapies for COPD have significantly improved the management of the disease, novel therapeutic approaches are still urgently needed, especially for suppressing inflammation in small airways (92). Cigarette smoke is one of the major factors for COPD and smoking cessation has so far been shown to diminish disease progression. Currently, several methods of nicotine replacement therapies are in use. However, the efficacy of these therapies remain to be further investigated (93). A variety of other interventions can lower the exacerbations in COPD, including influenza vaccination (94), anti-cholinergic bronchodilators (95), long-acting β_2_-agonists (LABAs) (96), and inhaled corticosteroids (ICSs). Further research is needed to understand the cell signaling mechanisms of COPD so that biomarkers can be identified and new therapies can be developed (92).

### Asthma

Asthma is a chronic inflammatory disease characterized by airway obstruction, excessive mucus production, and airway hyperresponsiveness (AHR). Asthma affects over 300 million people worldwide (97). The prevalence of asthma is increasing in many parts of the world, contributing to a substantial global economic burden. Clinically, asthma patients show episodes of cough, chest tightness, wheeze, and shortness of breath (55). There is currently no cure for asthma, but treatment can help ameliorate symptoms and improve the quality of life. Currently, the therapeutic challenge is to generate better inhalation technologies to improve the delivery of anti-inflammatory agents to the lung parenchyma (98). It was interesting to note that polymorphisms in antimicrobial proteins such as *β*-defensins can contribute to the severity of asthma. In fact, Baines et al., demonstrated that airway *β*-defensin-1 was elevated in severe asthma patients regardless of the inflammatory phenotype and may act as an effective biomarker and potential therapeutic target (88). Recent seminal work indicates that asthma is generally associated with Th2 immune responses, which is like other conditions such as atopic dermatitis. In this section of the review, we discuss the Th2 immune responses in asthma. Further, we discuss the available treatments and their limitations for asthma.

**Th2 immune responses in asthma:** The pathophysiology of asthma is complex and involves various host-environment interactions and inflammatory responses occurring at various levels. There are two subtypes of asthma: allergic and non-allergic. Allergic asthma is the most common form of disease that affects many individuals. Allergic asthma may get triggered in response to house dust mites, animal dander, mold, and pollens, whereas non-allergic asthma might be the result of respiratory viral infections, cold air, and tobacco smoke leading to chronic airway inflammation (99). Inflammation of the airways is a key component that leads to airway remodeling and damage. Th2 cytokines, including interleukin (IL)-5, IL-4, and IL-13 play a significant role in initiating the inflammatory cascade in asthma (100). A recent study showed that blockade of Th2-cell-mediated responses reduced symptoms for many patients, thereby demonstrating the central role of Th2 cells in the pathophysiology of asthma (101).

Eosinophils are bilobed leukocytes that are present in low numbers in healthy individuals. However, during asthma, Th2 cytokines such as IL-5 can induce prolonged eosinophil survival, activation, and trafficking to the site of injury (102). Recruitment of eosinophils to the site of injury results in an induction of cytokines and growth factors such as tumor growth factor b (TGF-β), leading to airway remodeling (103). An increased number of eosinophils have been observed in bronchoalveolar lavage (BAL) fluid and bronchial tissue in asthma patients (104). Further, elevated eosinophil counts correlate with disease severity, suggesting that these cells may play a significant role in asthma pathogenesis (104). IL-4 is a major cytokine in the development of inflammation in asthma. It is associated with the induction of isotype switching and secretion of IgE by B cells (105). IgE production, in turn, triggers the release of inflammatory cytokines, such as histamine, from mast cells that causes contraction of the smooth muscle in the airways, edema, and increased mucus secretion (106). Additionally, another crucial activity of IL-4 in stimulating inflammation is the induction of vascular cell adhesion molecule (VCAM)-1 on vascular endothelium in the asthmatic lung (107). Through the interaction of VCAM-1 and integrins on leukocytes, T lymphocytes, monocytes, and eosinophils can migrate to the injury site (108). Aberrant production of IL-4 due to genetic mutations or hyperresponsiveness to this cytokine might further contribute to the severity of asthma (109). Another cytokine that contributes to the pathophysiology of asthma is IL-13, a pleiotropic Th2 cytokine that contributes to goblet cell differentiation, activation of fibroblasts, and increased airway hyperresponsiveness (110). Further, IL-13 is believed to be a central regulator of mucus hypersecretion and fibrosis, leading to airway remodeling (111).

Most recently, a Th9 subtype of CD4^+^ T cells has emerged. These cells produce IL-9 a pleiotropic cytokine that may play role in inflammatory responses in asthma (83). IL-9 is secreted not only by CD4^+^ T cells but also by a variety of other T cells such as mast cells, and innate lymphoid cells (ILC-2) cells (112). It has been shown that in the presence of IL-4 and TGF-β, naïve CD4^+^ T cells develop into Th9 cells. In allergic diseases IL-9 has been shown to induce IgE production by B cells, activate mast cells, and play a pivotal role in mucus secretion (113, 114).

**Role of thymic stromal lymphopoietin in asthma:** Our understanding of the airway epithelium in driving asthma pathogenesis has progressed extensively in recent years. In response to allergic and non-allergic triggers, airway epithelial cells release cytokines, known as “alarmins,” such as TSLP, IL-33, and IL-25 (**Figure 2**), which stimulate downstream inflammatory responses (115). More recently, TSLP has emerged as an important factor in the pathogenesis of asthma, and increased levels of TSLP have been detected in asthma patients (116). TSLP can be induced by proinflammatory cytokines such as IL-1, TNF-α, bacterial and viral infections, Toll-like receptors, and allergens. The nuclear factor-kappa-B (NF-kB) pathway plays an important role in the mechanism of TSLP activation (117). Once TSLP is upregulated, it binds to its receptor TSLPR (118). A recent study showed that IL-7Rα binds to the TSLP/TSLPR complex with high affinity (119), and this ternary complex initiates a signaling cascade that activates STAT5 transcriptional factors (120). TSLP can interact with dendritic cells (DCs) to enhance antigen presentation to induce CD4^+^ cells (121). TSLP-activated mature DCs can then migrate to lymph nodes to initiate adaptive responses. Further, during non-allergic asthma, TSLP activates innate lymphoid cells (ILC-2) to produce Th2-like cytokines (121). According to a recent study, the blockade of TSLP reduces allergen-induced inflammation in animal models (122).

**Current treatment options for severe asthma:** Multiple phenotypes of severe asthma have been identified, showing the significance of tailored treatment options to meet individual needs. Most patients with asthma attain decent control of the disease with the use of inhaled corticosteroids and bronchodilators (123) However, many patients with asthma, especially in developing countries, remain unattended and suffer from poor quality of life (124). Recently, monoclonal antibodies have emerged as a new treatment option for uncontrolled asthma, targeting different signaling molecules in the pathways causing airway inflammation. Omalizumab is the first FDA-approved drug to treat severe asthma (125). It binds to IgE and prevents binding to its receptor on mast cells (125). Mepolizumab is a fully-humanized anti–IL-5 antibody that binds to IL-5 with high affinity to prevent IL-5 from binding to IL-5 receptors on eosinophils (126). Although many treatment options are available for patients with asthma, not all people with severe disease can benefit from the same biological drug, and therefore, an appropriate biological drug must be carefully selected for each patient. Although management of asthma varies among individuals, it’s crucial to protect patients with asthma from infectious lung diseases caused by bacteria and viruses. People with asthma are more susceptible to developing pneumonia due to previous lung damage. The section below explains several lung infectious diseases such as bronchitis, pneumonia, and disease pathogenesis.

## Lung infection

The etiology of lung infections generally involves the acquisition of a pathogen, its dissemination, and invasion of the underlying respiratory tract tissue. Respiratory tract infections are classified based on their symptomatology and anatomic involvement. Upper respiratory tract infections are usually benign, transitory, and self-limiting. These are caused by viruses, bacteria, mycoplasma, and fungi (**Table 3**), mostly resulting in the common cold, epiglottitis, laryngitis, pharyngitis (sore throat), or sinusitis (sinus infection). On the contrary, lower respiratory tract infections are predominantly caused by bacteria, mycoplasma, viruses, and sometimes fungi (**Table 3**). These result in bronchitis, bronchiolitis, and pneumonia, which can be severe to fatal.

**Table 3:** Infectious diseases of the Lung.

Clinical Illness	Causative Agent		Pathogen Recognition Receptor implicated	Ref
	Agent	Examples		
**Upper Respiratory Tract Infections**				
Common Cold	Virus	Rhinovirus	ICAM-1, CDHR3, SP-A, SP-D	(127–129)
		Influenza virus	Sialic acids, Ganglioside GM-3, RIG-I, SP-A, SP-D	(129–132)
		Coronavirus	ACE2, TMPRSS2, BSG (CD147) and FURIN	(133)
		Respiratory Syncytial Virus	TLR3, SP-A, SP-D	(133)
	Bacteria	Rare		
	Fungi	Rare		
	Other	Rare		
Pharyngitis	Virus	Adenovirus	CAR, Integrins, Heparan sulfate and Sialic acids	(134, 135)
		Rhinovirus	ICAM-1, CDHR3, SP-A, SP-D	(127–129)
		Influenza virus	Sialic acids, Ganglioside GM-3, RIG-I, SP-A, SP-D	(129–132)
		Coronavirus	ACE2, TMPRSS2, BSG (CD147) and FURIN	(133, 133)
		Epstein-Barr virus	HLA-DR, CR-1 and CR-2	(136–139)
		Herpes simplex virus	HveA, HveB and HveC	(140, 141)
	Bacteria	Group A Hemolytic Streptococci	TLR-2, IL-1R, Sialic acids	(142–144)
		*Corynebacterium diphtheriae*	TLR-2, TLR-9, Mincle	(144)
		*Haemophilus influenzae*	TLR-2, 4, 5, and 9, SP-A, SP-D, PAFR	(145, 146)
		*Streptococcus pneumoniae*	TLR-2, TLR-4, MACRO, SRA-I, SRA-II, DC-SIGN, CRP, MBL, PAFR	(147, 148)
		*Mycoplasma pneumoniae*	TLR 1, 2, 4 and NOD-2	(149)
	Fungi	*Candida albicans*	Mannose receptor, TLR4, Dectin-1	(150)
	Other	Rare		
Epiglotitis	Virus	Respiratory Syncytial Virus	TLR3, SP-A, SP-D	(129, 151)
		Parainfluenza virus	Sialic acids, Heparan sulfate, NKp-46	(152, 153)
	Bacteria	*Haemophilus influenzae*	TLR-2, 4, 5, and 9, SP-A, SP-D, PAFR	(145, 146)
		*Corynebacterium diphtheriae*	TLR-2, TLR-9, Mincle	(144)
	Fungi	Rare		
	Other	Rare		
**Lower Respiratory Tract Infections**				
Bronchitis	Virus	Adenovirus	CAR, Integrins, Heparan sulfate and Sialic acids	(134, 135)
		Rhinovirus	ICAM-1, CDHR3, SP-A, SP-D	(127–129)
		Respiratory Syncytial Virus	TLR3, SP-A, SP-D	(129, 151)
		Influenza virus	Sialic acids, Ganglioside GM-3, RIG-I, SP-A, SP-D	(129–132)
	Bacteria	*Haemophilus influenzae*	TLR-2, 4, 5, and 9, SP-A, SP-D, PAFR	(145, 146)
		*Streptococcus pneumoniae*	TLR-2, TLR-4, MACRO, SRA-I, SRA-II, DC-SIGN, CRP, MBL, PAFR	(147, 154)
		*Moraxella catarrhalis*	TLR-2, 4 and 9 CEACAM1	(155, 156)
		*Mycoplasma pneumoniae*	TLR 1, 2, 4 and NOD-2	(149)
	Fungi	Rare		
	Other	Rare		
Bronchiolitis	Virus	Adenovirus	CAR, Integrins, Heparan sulfate and Sialic acids	(134, 135)
		Rhinovirus	ICAM-1, CDHR3, SP-A, SP-D	(127–129)
		Respiratory Syncytial Virus	TLR3, SP-A, SP-D	(129, 151)
		Parainfluenzae virus	Sialic acids, Heparan sulfate, NKp-46	(152, 153)
		Herpes simplex virus	HveA, HveB and HveC	(140, 141)
	Bacteria	*Haemophilus influenzae*	TLR-2, 4, 5, and 9, SP-A, SP-D, PAFR	(145, 146)
		*Streptococcus pneumoniae*	TLR-2, TLR-4, MACRO, SRA-I, SRA-II, DC-SIGN, CRP, MBL, PAFR	(147, 154)
	Fungi	Rare		
	Other	Rare		
Pneumonia	Virus	Adenovirus	CAR, Integrins, Heparan sulfate and Sialic acids	(134, 135)
		Influenza virus	Sialic acids, Ganglioside GM-3, RIG-I, SP-A, SP-D	(129–132)
		Respiratory Syncytial Virus	TLR3, SP-A, SP-D	(129, 151)
		Parainfluenzae virus	Sialic acids, Heparan sulfate, NKp-46	(152, 153)
		Coronavirus	ACE2, TMPRSS2, BSG (CD147) and FURIN	(133, 133)
		Varicella zoster virus	Heparan sulfate	(157)
		Measles virus	SLAMF-1, PVRL-4 and DC-SIGN	(158)
		Herpes simplex virus	HveA, HveB and HveC	(140, 141)
	Bacteria	*Streptococcus pneumoniae*	TLR-2, TLR-4, MACRO, SRA-I, SRA-II, DC-SIGN, CRP, MBL, PAFR	(147, 154)
		*Staphylococcus aureus*	TLR-2, 9, EGFR, TNFR-, IFNAR	(159)
		*Haemophilus influenzae*	TLR-2, 4, 5, and 9, SP-A, SP-D, PAFR	(145, 146)
		*Klebsiella pneumoniae*	TLR-2, 4, EGFR, SP-A, SP-D	(160)
		*Pseudomonas aeruginosa*	aGM-1, TLR-2,4 and 5, CFTR	(161)
		*Mycoplasma pneumoniae*	TLR 1, 2, 4 and NOD-2	(149)
		*Legionella pneumophila*	Naip5, Ipaf	(162)
		*Mycobacterium tuberculosis*	TLR 2, 4, 9, Dectin-1, Mannose receptor, NOD-2, DC-SIGN	(163)
		*Coxiella burnetii*	TLR4, Integrin receptors	(164)
		*Chlamydia pneumoniae*	TLR4, NOD-1 and NOD-2	(165, 166)
	Fungi	*Histoplasma capsulatum*	Dectin-1	(167)
		*Candida albicans*	Mannose receptor, TLR4, Dectin-1	(150, 168)
		*Aspergillus fumigatus*	Pentraxin 3	(169)
	Other	*Pneumocystis (carinii)* *Jirovecii*	Manose receptor, Dectin-1	(170)

### Bronchitis and bronchiolitis

**Etiology:** Bronchitis and bronchiolitis involve inflammation of the bronchial tree. Bronchitis is usually preceded by an upper respiratory tract infection and can be caused by a combination of environmental factors and bacterial infections by pathogens such as *Haemophilus influenzae* and *Streptococcus pneumoniae*. Chronic bronchitis is classically defined as persistent cough and sputum production with periodic exacerbation (171). Bronchiolitis is a viral respiratory disease of infants and is caused primarily by respiratory syncytial virus (RSV), influenza viruses, or rhinovirus. Bronchiolitis is a mild, self-limited infection in most children but may sometimes progress to respiratory failure in infants (172).

**Pathogenesis and clinical manifestations:** When the bronchial tree is infected, inflammation ensues, and the mucosa becomes hyperemic and edematous. Bronchial secretion increases in the mucosa and mucociliary function of the epithelium reduces. This reduces lower respiratory volume and leads to increased dyspnea, tachypnea, large amounts of sputum, tightness in the chest, fatigue, and confusion (173). The extent of damage to the mucosa can depend on the organisms(s) involved, bacterial toxins secreted, and other metabolic products produced (174). Infants with bronchiolitis suffer from inflammation in the airways and necrosis of the respiratory epithelium. Bronchial and bronchiolar walls thicken in bronchiolitis, and an exudate made up of necrotic material and respiratory secretions narrow the bronchial lumen, leading to airway obstruction. Clinical manifestations include fever, deepening of cough, dyspnea, tachypnea, wheezing, or actual lack of breath. Areas of air-pockets can develop, which may eventually result in respiratory failure and death (172, 174).

**Microbiological diagnosis:** Cough in the absence of fever, tachycardia, and tachypnea suggests bronchitis; however, bacteriological examination and culture of purulent respiratory secretions must be done for confirmation (175). Rapid diagnostic tests can be used to detect several pathogens linked to acute bronchitis; however, not all rapid tests are widely available and cost-effective. Aspirations of nasopharyngeal secretions or swabs from infants with bronchiolitis are collected as samples and serologic tests are done to identify the virus (176).

**Prevention and treatment:** With only a few exceptions, viral infections are treated with symptomatic care. Children with mild-to-moderate symptoms can be treated with nasal saline, antipyretics, and a cool-mist humidifier. On the contrary, children with severe respiratory distress should be admitted to the hospital and carefully monitored. The use of corticosteroids, bronchodilators, or prophylactic measures may benefit children with chronic bronchitis. RSV infections in infants may be treated with ribavirin, and influenza type A viruses may be treated with amantadine and rimantadine (175).

### Pneumonia

Pneumonia is an inflammation of the lung parenchyma, caused by several factors such as environmental contaminants, autoimmune diseases, and infection. Anatomically, pneumonia can be classified as lobar pneumonia (affecting the entire lobe) or bronchopneumonia (affecting patches of the lobe). Further, it can be etiologically classified as community-acquired pneumonia (CAP, infection in a previously healthy individual) or hospital-acquired pneumonia (infection in a hospitalized individual within 48 hours of admission).

**Etiology:** *Bacterial pneumonias- S. pneumoniae* is the most common cause of CAP and a leading cause of illness in children below 2 years of age, the elderly, and immunocompromised individuals (177). Pneumonias caused by other *Streptococci* are uncommon. *Streptococcus pyogenes* pneumonia is often associated with hemorrhagic pneumonitis and empyema. Pneumonia due to *H. influenzae* (usually nontypable) and *Klebsiella pneumoniae* are more common among patients over 50 years old with chronic obstructive lung disease or alcoholism, whereas pneumonia due to *Mycoplasma pneumoniae* and *Chlamydophila pneumoniae* are more common in school-aged children. Other less common agents causing pneumonias include *Francisella tularensis*, *Legionella pneumophila*, *Yersinia pestis*, and *Neisseria meningitidis* (178). *Mycobacterium tuberculosis* (Mtb) causes pulmonary pneumonia; however, the incidence of tuberculosis is low in industrialized countries. Nonetheless, it remains to be a significant public health problem in the United States.

*Viral pneumonias-* Pneumonias by RSV and Adenoviruses are rare in the healthy population. Pneumonia caused by influenza viruses is still a cause of high mortality in the elderly and in patients with underlying diseases. A serious complication of influenza is a secondary bacterial pneumonia which can be fatal in infants.

*Other pneumonias- Actinomyces* and *Nocardia* spp can cause pneumonitis. *Aspergillus* and *Candida* spp cause pneumonias in severely ill or immunosuppressed patients and neonates, whereas *Pneumocystis carinii* causes life-threatening pneumonia in patients with HIV.

**Pathogenesis and clinical manifestations:** The onset of pneumonia is quite abrupt; it develops with a shaking chill, fever, malaise, cough, and dyspnoea. Left untreated, this toxic illness can progress to acute respiratory failure, septic shock, multiorgan failure, and death within several days from onset (179). Respiratory failure due to pneumonia occurs when inflammatory exudate fills the alveoli and reduces its normal functional capacity. The persistence of pulmonary artery blood flow to the consolidated lung airspace during pneumonia leads to an intrapulmonary shunt that can also result in respiratory failure (180).

**Microbiological diagnosis:** A clinical examination of sputum is done to identify the infectious agent. Cultures of sputum, blood, and pleural fluid help in identifying the causative agent. The sputum quellung test can be used to identify *S. pneumoniae, H. influenzae*, and *K. pneumoniae* by serotype. In addition, rapid diagnostic tests based on PCR, ELISA, or other DNA probes can be used to identify other causative agents of pneumonia. Viral infections are usually diagnosed by serological testing for related antigens or antibodies in pulmonary secretions.

**Prevention and treatment:** Clinical diagnosis of the pathogen causing pneumonia is of utmost importance to deploy effective treatment options. Amoxicillin or amoxicillin clavulanate is the most common first-line antimicrobial agent used for CAP (181). In children suspected of having influenza and moderate-to-severe CAP, early antiviral therapy has been proven to be very helpful (182). Pneumococcal vaccines are available for high-risk populations; and along with yearly influenza vaccinations have been proven to reduce pneumonia burden and hospitalizations in children and adults (176). However, due to the presence of allelic variations amongst the bacterial serotypes, the current vaccines do not provide protection against all serotypes (183, 184). Since transmission occurs by droplets or contact, good hand washing, and good personal hygiene are the most important measures to prevent the spread of pneumonia.

## COVID-19

COVID-19 is a viral infection caused by coronavirus SARS-CoV-2 that can progress to severe acute respiratory syndrome with pneumonia and acute respiratory distress syndrome (185). In 2019 the disease spread rapidly and became a pandemic affecting more than 600 million people worldwide. Histologically, COVID-19 shows diffuse alveolar damage corresponding to the phase of the disease (acute to fibrotic) and can be divided into 3 main injury patterns: epithelial, vascular and fibrotic affecting the upper respiratory tract in mild disease and bilateral lobes of the lung in more severe disease. The etiology of the disease includes attachment of SARS-CoV-2 virus to target cells *via* angiotensin converting enzyme-2 (ACE-2) receptors that promotes release of viral RNA into the host cell, initiating replication and further dissemination (186). Clinical features of the disease include fever, dry cough, shortness of breath, fatigue, myalgias, nausea or headache and weakness that may further develop into pneumonia, ARDS or death as the pathology worsens (187).

The pathophysiology involves diffuse or spotty changes in lung parenchyma that turn edematous due to congestion, punctuate hemorrhages, and hemorrhagic necrosis, particularly at the peripheral edges of the pulmonary lobe. Bronchi and bronchioles may show chronic inflammation with marked thickening of bronchial mucosa due to edema. The peripheral or central pulmonary arterial vessels are filled with platelet-fibrin thrombi and inflammatory cells and may result in fibrinoid necrosis, wall thickening, luminal stenosis, or occlusion, leading to the development of pulmonary hypertension in later stages in some critical patients (188). The clinical diagnosis is based on detection of viral RNA by RT-PCR in Nasopharyngeal swabs or sputum samples. The treatment guidelines recommend home management for patients with mild symptoms and hospital care for patients with severe disease requiring oxygenation support. New age mRNA-based vaccines along with viral vector based or whole inactivated viral vaccines are already being prescribed as a strategy to manage viral transmission (189).

## Application of lung immunology in drug development

To treat lung-related pathologies, the delivery of drugs and vaccines through the nasal and upper respiratory pathway is required. The ongoing COVID-19 pandemic involves a virus that causes significant damage to the lung. There have been several therapies and vaccines that treat and prevent disease progression; however, there is very limited development in delivery to the lung. In the following section, we have reviewed various formulations, devices, and human factors that contribute to the development of upper respiratory tract therapies. We have further compared the requirements for reaching the nasal mucosa, upper respiratory, and mid respiratory tract with those for deep lower respiratory tract.

Drug delivery through the respiratory route is a major strategy to treat asthma and various lung diseases. The devices for delivery of aerosols to treat respiratory diseases have been improvised over several decades from the classical models of nebulizers and pressurized metered-dose inhalers to a myriad of inhaler designs and devices, including dry powder inhalers, soft mist inhalers, and smart nebulizers. Educating and training on the proper use of these devices, determining the suitability of the device type and design according to the personal ease and convenience of the patient, and close monitoring of patient recovery following the use of appropriate inhaler device and the type of formulation are crucial to derive maximum health benefits from the administration of medications using these devices. Delivery of drugs directly to the airways of the respiratory tract and lungs can help reduce the dosage of medication required and improve the effectiveness of the therapy as compared to oral administration of drugs or systemic administration into the bloodstream of the patient.

### Physicochemical properties of aerosolized drugs

The effective treatment of respiratory disorders using inhalation devices is closely associated with the formulations available for different types of diseases and the efficiency of the inhaler to aerosolize and deliver the drug to the appropriate location in the respiratory tract. For example, albuterol and ipratropium are bronchodilators used to treat COPD. These are available as liquid formulations (solutions) that can be conveniently aerosolized and delivered to the patient using nebulizers and pressurized metered-dose inhalers (pMDI) (190–193). Three processes govern the deposition of aerosol particles in the respiratory tract: inertial impaction, sedimentation, and diffusion. For most of the inhaled medications, the principal mechanisms of aerosol deposition are impaction and sedimentation (194, 195). Particles of size 3-7 µm get deposited by impaction predominantly in the oropharynx and the larger conduits of the respiratory tract. This impaction takes place particularly at the bifurcation of airways and sites, posing an obstruction to free flow of air, whereas the smallest aerosol particles (2-3 µm) can reach the terminal bronchioles and alveoli (194–196). Aerosol particle sedimentation is influenced by several factors including gravity, dimensions of air conduits, and breath hold time. The fine-particle fraction (FPF) of the inhaled drug that contains <5 µm aerosol particles is most crucial in bringing about desired clinical effects. Inhalers differ widely in respect to the fine-particle fraction, ranging from 10%–50% for pMDI, 12%–35% for dry powder inhalers (DPI), to 30%–50% for the soft mist inhalers (SMI).

The physicochemical and pharmacokinetic properties of drugs, such as half-life, molecular weight, solubility, lipophilicity, charge, and protein binding are important in determining their clearance from the lungs (8). Lipid-soluble molecules can traverse the respiratory tract epithelium by passive transport, whereas hydrophilic molecules travel through extracellular conduits. Medications administered by inhalation are deliberately rendered lipophilic to allow slow dissipation from the lungs and thus exert their effect over a longer duration.

### Drug inhalation devices

**Figure 3:**
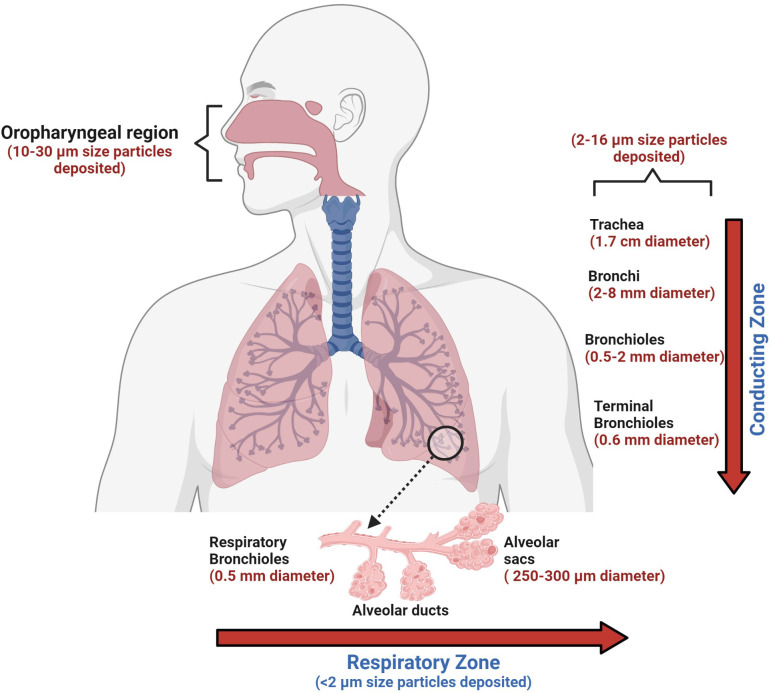
The human conducting and respiratory zones. The conducting and respiratory zones include the nasal cavity, pharynx, larynx, trachea, bronchi, bronchioles, and alveolar sacs. The air-conducting zones become smaller and smaller as the airways branch out into smaller tubes. Trachea, a 10 cm long structure with the largest diameter, is made of cartilage rings that provide structural support to the upper respiratory region. As the trachea branches into bronchi and further into smaller bronchioles, the inner epithelial lining also changes from thicker, taller, and ciliated cells to a thinner epithelium which has shorter and non-ciliated cells. The thinner lining promotes the exchange of inhaled air into the bloodstream in the alveolar sacs. Orchestrated movement of cilia in the tracheal region, faster air currents, and mucus efficiently trap particles larger than 10 microns, contrary to particles less than 2 microns. Particles that are less than 2 microns have higher chances to reach alveoli, which can lead to impaired gas exchange, which may result in serious health consequences.

The choice and suitability of an aerosol delivery device for a particular patient depends on the recommendations of the physician, available types of formulations, and compliance with the personal ease and comfort of the patient (**Table 4** and **Figure 3**).

**Table 4:** Comparison of inhalers.

Device	Advantages		Limitations
Pressurized metered dose inhaler	• Portable • Compact • Multidose device • Dose delivered and particle size relatively independent of inhalation maneuver • Quick and easy to use for many patients • Suitable for emergencies • Available for many formulations • Not breath-actuated		• Requires coordination of inspiration and actuation • Not suitable for young children (without use of a valved holding chamber) • High oropharyngeal deposition (without use of a valved holding chamber) • Some have no dose counter • Propellant required • Need to shake vigorously prior to use • Need to prime if not used recently • Not all medications are available
Dry Powder Inhaler	• Portable • Compact • Breath-actuated • Less coordination needed • Short treatment time • Available for many formulations • Dose indicator		• Moderate to high inspiratory flow required for most devices • Not suitable for young children • Some devices are single-dose • May not be suitable for emergencies • Some devices are susceptible to environmental humidity • Inability to use with a valved holding chamber • Not all medications are available
Soft Mist Inhaler	• Portable • Multi-dose device • Less dependence on inspiratory flow • Slow velocity aerosol • High fine-particle fraction and relatively • high lung deposition • Long plume duration • Less coordination needed • No propellant • Dose indicator • Does not require a spacer (in those > 5 yrs old) • Suitable for use in children		• Device needs to be assembled initially • Not breath-actuated • Need to prime if device not used within last several days • Not all medications are available
Jet Nebulizer	• Less coordination needed • Effective with tidal breathing • High doses can be easily administered • Dose modification possible • Combination therapy if drugs compatible • Some are breath-actuated		• High cost • Less portable • Pressurized gas source required • Lengthy treatment time • Contamination possible • Device preparation required • Not all medications available
Ultrasonic Nebulizer	• Less coordination needed • High doses can be given • Small dead volume • Quiet • Faster delivery than jet nebulizer • Less drug loss during exhalation • Some are breath-actuated		• High cost • Need for electrical power • Contamination possible • Prone to malfunction • Possible drug degradation • Does not nebulize suspensions well • Device preparation required • Potential for airway irritation • Not all medications available
Mesh Nebulizer	• Less coordination needed • Effective with tidal breathing • High doses can be easily given • Dose modification possible • Some are breath-actuated • Small dead volume • Quiet • Faster delivery than jet nebulizer • Less drug loss during exhalation • Portable and compact • High dose reproducibility		• Cost • Contamination possible • Device preparation required • Not all medications available

**pMDIs** were the foremost among the extensively used inhaler devices (190, 191, 197) and continue to be popular to date due to ready availability, reasonably low cost, multidose drug administration, small size, easy portability, and the availability of many compatible formulations. The majority of the patients with asthma or COPD are administered with bronchodilators (albuterol/ipratropium) using pMDIs. Inhaled drugs for the treatment of obstructive lung disorders are widely available for use with pMDI, either singly or in combination. The formulation used in PMDIs may be a solution, suspension, or co-suspension. In addition, it contains other ingredients like propellants, co-solvents, and surfactants (for example, lecithin or oleic acid) (198). Sometimes, pMDIs show a problem of flocculation of drug crystals, leading to sedimentation. Particle crystal agglomeration can be minimized using surfactants. A novel strategy, known as co-suspension technology, helps to ameliorate this problem. It involves attaching phospholipid particles to the drug molecules to render a more uniform drug suspension, enabling the formulation of drug combinations and relying less on the need for shaking before use (199). pMDIs are not suitable for inhaled drug administration to young children without the use of a valved holding chamber. Moreover, administration using pMDIs results in high deposition of the drug in the oropharynx with relatively less amount reaching the finer airways in the lungs. The requirement of a propellant and the need to shake vigorously before use are some other concerns pertaining to the use of pMDIs.

**Nebulizers** are the earliest among the modern methods of delivering aerosols to the lungs for respiratory drug delivery. Nebulizers are routinely used in hospitals and home settings. Several newer nebulization technologies have enabled more efficient use of nebulizers as drug delivery devices. A jet nebulizer harnesses a stream of compressed gas to generate droplets from the drug solution or suspension (200). Some conventional jet nebulizer designs (like non-vented jet nebulizers) result in substantial loss of the aerosolized drug into the room air. This problem has been circumvented by the development of mesh nebulizers and ultrasonic nebulizers. Mesh nebulizers can produce droplets of uniform size without imposing high mechanical stress on the drug solution, they are associated with relatively less temperature rise during the nebulization process (201), and they can administer higher drug doses leading to faster patient recovery (202), they do not require baffles, thus minimizing residual losses, and they can nebulize a myriad of solutions and suspensions. These devices operate using batteries or a direct power supply, eliminating the requirement of an external gas flow. However, mesh nebulizers have some issues associated with them. They are costlier than jet nebulizers. The apertures in the mesh are prone to clog, and the high surface area of the mesh (owing to the presence of numerous apertures) requires careful cleaning (202). Examples of FDA-approved drugs for delivery using mesh nebulizers include glycopyrrolate (a long-acting muscarinic antagonist for COPD) and aztreonam (an antibiotic for cystic fibrosis).

**Ultrasonic nebulizers** are electrically powered devices in which a drug solution is subjected to ultrasonic waves of high frequency, resulting in an aerosol generation. Portable, small-sized ultrasonic nebulizers are available in the market for the administration of bronchodilators. They are more expensive than jet nebulizers but are comparable to mesh nebulizers in this regard. Treprostinil (available for inhalation under the brand name Tyvaso) has been successfully used to treat patients with pulmonary arterial hypertension using an ultrasonic nebulizer (203). A major drawback of ultrasonic nebulizers is the possible drug inactivation due to ultrasonic waves. Moreover, they are not efficient in nebulizing a suspension (204).

**Soft mist inhalers (SMIs)** (Respimat, Boehringer Ingelheim, Ingelheim am Rhein, Germany) were originally available for the administration of ipratropium/albuterol, which later became available for other bronchodilators (205, 206). A compressed spring within the device pushes the drug through a membrane, emitting an aerosol (205). It is not necessary to shake before using the device. It is a portable, multi-dose device that generates aerosols with a high fine-particle fraction, resulting in substantial deposition of drugs in the lungs. In comparison to pMDIs, Respimat discharges aerosols slower (205, 206) and over a longer duration (-1.2 s as compared to < 0.5 s from pMDIs) which may reduce the coordination required for actuation and inspiration, thus improving the likelihood for greater lung deposition. Further, it does not require a propellant and is suitable for use in children.

**Dry powder inhalers (DPIs)** are excellent alternatives to pMDIs as they help to circumvent many limitations associated with pMDIs such as low lung deposition of drugs and the need for propellants. In 1967, Fisons (Ipswich, UK) launched the Spinhaler^®^ device. Thereafter, striking advances have been made in DPI design. and a wide variety of medications are available as dry powder formulations, making them extremely useful to patients with respiratory tract diseases. Some of the commercially available DPI designs are Genuair, Breezhaler, Podhaler, Dreamboat, Conix, Twincer, Twincaps, and Staccato. They are compact and easily portable and can help impart short treatment times. The TOBI^®^ Podhaler^®^ (Novartis, Basel, Switzerland) holds merit over jet nebulizers for the treatment of cystic fibrosis. Administration of Tobramycin Inhalation Solution (TIS™) (Novartis, Basel, Switzerland) using a jet-nebulizer posed several problems. For example, the device requires long inhalation times cleaning of the nebulizer is required after every use, which increases the chances of lung infection from devices that are not cleaned properly (207). Moreover, nebulizers are rather voluminous and require a jet of compressed air to generate aerosols. Further, TIS should be refrigerated during storage (208), and the product may undergo degradation before use. Considering these factors, the TOBI Podhaler is safer and more convenient for cystic fibrosis patients.

However, there are some demerits of DPIs. Due to inter-particulate forces like electrostatic interactions and van der Waals forces (209), micronized powders are adhesive/cohesive and spontaneously form agglomerates. These agglomerates must be deagglomerated prior to or during the processes of aerosolization and inhalation to produce a substantial fraction of particles within the range of 1–5 µm in size. These particles can reach small tubules and alveolar spaces in the lungs. In passive DPIs, the energy required to overcome the above-mentioned inter-particulate forces is derived from the patient’s inspiratory flow (210, 211), whereas, in active DPIs, energy is harnessed *via* other sources. The merit of a passive DPI is that it averts the need to synchronize the actuation and inspiration maneuver by the patient. However, this requires a pronounced inspiratory effort on the part of the patient, which might be difficult for the elderly and children, and those suffering from COPD or asthma (212). Because the rheological properties of micronized powders are often poor, in most formulations, drug particles are blended with larger carrier particles (30–90 μm in size) such as lactose to facilitate deagglomeration and powder flow (213). The performance of DPIs is susceptible to environmental humidity as dry powder formulations might absorb moisture from the environment. Further, these devices are not amicable to use with valved holding chambers.

A wide variety of inhaler devices are commercially available today; however, knowledge of correct device use, device maintenance, and stringent compliance to the instructions provided by the medical practitioner are pivotal in achieving maximum and timely therapeutic benefits. Newer models of inhalers are constantly on the horizon to make them more user-friendly and convenient. Effective communication and coordination among doctors, nurses, and patients and being updated with the latest developments in the field of inhaler device designs and applications can help in the effective and timely implementation of treatment regimens.

## Exosomes as drug delivery vehicles in lungs

Exosomes are naturally occurring nanoparticles present in most bodily fluids such as serum, plasma, urine, breast milk, saliva, ascites, and synovial and amniotic fluids. These extracellular vesicles are like bacterial outer membrane vesicles and vary in size from 20 microns to 200, assisting in cell-cell communication and intercellular transport (17, 214). The exosomes contain exogenous biomolecules such as mRNA, proteins, DNA, miRNA, lipids and conserved transmembrane proteins such as CD81, CD63, and CD9, which are used as biomarkers for identifying exosomes. As the expression of these transmembrane proteins is based on cell type, they are responsible for delivering the cargo by identifying the specific recipient cells (17, 18). Several studies have demonstrated the advantages and ability of these nanocarriers over synthetic liposomes for drug delivery (18, 19). Current bioengineering techniques permit modification of exosome surface to enhance cargo loading, allowing superior drug delivery to targeted cell types. Even though biological nanoparticles have been investigated as drug delivery vehicles for several diseases, using exosomes for treating pulmonary disorders is still in its early phase. Recently Kim et al. developed an engineered exosome with therapeutic peptides and curcumin, which can be delivered into the lungs by inhalation. These engineered exosomes significantly reduced proinflammatory cytokines and inhibited inflammation (19). In another study by Popowski et al., lung-derived exosomes were used as inhaled nanoparticle delivery systems, which delivered mRNA and protein drugs. As these exosomes are delivered by inhalation and are naturally derived, the pulmonary bioavailability of the encapsulated drugs increases significantly (22). The drug must reach the deep lung tissue for treating lower respiratory diseases, and the pulmonary distribution should be optimal. Drug-carrying exosomes fulfill all these criteria and are naturally optimized molecules that can be delivered to the targeted site using currently available techniques. However, further investigation and clinical studies need to be directed to completely understand the long-term health effects of these biological nanoparticles. Further, more studies are required to understand their full potential as drug delivery systems.

## Conclusion

The normal functioning of the lung requires complex interactions of many different cell types embedded in a precise architecture of the lung. Normal human lungs are constantly exposed to a plethora of inflammatory molecules, allergens, pollutants, and microbes from the environment and can induce cough, shortness of breath, chest pain, and fever. The nature of the immune responses to these foreign entities involves a coordinated action of the innate and adaptive immunity, which enables a balanced response and minimize lung pathology.

In this review we have described various aspects of lung homeostasis and innate and adaptive responses required to maintain a balanced state. We reviewed lung inflammatory responses and infectious diseases contributing to lung disease pathogenesis with a brief description of COVID-19 lung pathology. Further, we reviewed currently available therapies to treat lung diseases and devices used to deliver drugs including exosomes, directed to the airways of the respiratory tract. The review provides a guide to lung immunology expanding on the interplay of different aspects of the immune system, inflammation and lung associated infectious diseases and how various strategies are used for lung pathology treatment. It aims to provide a technical insight into the current landscape of drugs and devices used to treat various lung diseases and discusses their limitations to initiate a colloquy about future targets of therapeutic interventions.

## Author contributions

IG: Wrote the Lung Inflammation Section; RD: Wrote the Infectious Diseases Section; VB: Wrote the Therapy and Devices Section; VG: Wrote the Lung Immunology and Exosomes as drug delivery Section; NC: Conceived the concept and directed the writing. All authors contributed to the article and approved the submitted version. IG, RD and VB contributed equally to the manuscript.
